# Photodetection Characteristics of Gold Coated AFM Tips and n-Silicon Substrate nano-Schottky Interfaces

**DOI:** 10.1038/s41598-019-49908-1

**Published:** 2019-09-19

**Authors:** Yawar Abbas, Ayman Rezk, Irfan Saadat, Ammar Nayfeh, Moh’d Rezeq

**Affiliations:** 1 Department of Physics, Khalifa University, Abu Dhabi, 127788 UAE; 2 Department of Electrical Engineering and Computer Science, Khalifa University, POB 127788 Abu Dhabi, UAE

**Keywords:** Nanoscience and technology, Optics and photonics, Physics

## Abstract

Silicon (Si)-based photodetectors are appealing candidates due to their low cost and compatibility with the complementary metal oxide semiconductor (CMOS) technology. The nanoscale devices based on Si can contribute efficiently in the field of photodetectors. In this report, we investigate the photodetection capability of nano-Schottky junctions using gold (Au) coated conductive atomic force microscope (C-AFM) tips, and highly cleaned n-Si substrate interface. The Au nanotip/n-Si interface forms the proposed structure of a nano Schottky diode based photodetector. The electrical characteristics measured at the nanoscale junction with different Au nanotip radii show that the tunneling current increases with decreasing the tip radius. Moreover, the tunneling process and photodetection effects are discussed in terms of barrier width/height decrease at the tip-semiconductor interface due to the applied electric field as well as the generation of plasmon-induced hot-electron at the nanoparticle (i.e. C-AFM tip)/n-Si interface. Furthermore, the photodetection sensitivity is investigated and it is found to be higher for C-AFM tips with smaller radii. Moreover, this research will open a new path for the miniaturization of photodetectors with high sensitivity based on nano-Schottky interfaces.

## Introduction

The optoelectronic characteristics of nanostructures (e.g. nanowires, quantum dots and nanosheets), have been investigated^1–5^, and these in turn revealed the fascinating features of nanomaterials. Owing to their excellent response for the light radiations, in terms of dynamic range and sensitivity, these nanostructures placed themselves as outstanding candidates for the building blocks of new generation of photodetectors^6–9^. The effect of light irradiation on the electrical characteristics of nanostructure based photo-sensors have been investigated extensively. These are powered by either external power source, which is utilized to spur photogenerated carriers to generate photocurrent, or by novel self-powered photodetectors that work without external power source^10–14^. Irrespective of the detection techniques, generally the fundamental operation of the photodetector depends on the transition of electron from lower energy to higher energy states as a result of absorption of photon. This process, in turn, manifests as an increase in the magnitude of electric current during the electrical measurements of these devices.

Due to their compatibility with complementary metal oxide semiconductor (CMOS) processing, germanium (Ge) or Si based photodetectors have been reported^15–19^. To reduce reflectance and enhance absorption, laser etched micro-structured Si-based photodetectors have been developed^20^. Most of the conventional photodetectors are investigated based on the photo absorption of the materials that are fabricated for the purpose of photosensors^21–24^. For scaling down of the semiconducting devices, with lower power consumption, the investigation of photodetection at the nano-scale metal-semiconductor (MS) interface is of great significance^25–27^.

The conductive-probe atomic force microscopy (C-AFM) has been used as a powerful tool to investigate the photodetection at the nano-scale MS junctions^28–30^. The main advantage of C-AFM electrical measurement is its ability to gather local conductivity information^31–33^. Due to the very small size of the nanotip, one can detect the effect of light on the tunneling current from the nanotip to substrate due to narrowing of the energy band gap width by the enhancement of local electric field at the nanotip-substrate interface^34^.

Studies revealed that for nanoscale Schottky diodes, the Schottky barrier height (SBH) becomes a function of the diode size. Consequently, the contribution of the tunneling current to the total conductance greatly enhances^35^. The effect of Au NPs at Pd/SiC interface is also studied and it is concluded that the effect of SBH increases with the mean size of Au-NPs^36^. The CAFM measurements further reassured that the size of nano-Schottky contacts at nanoscale prominently affects the behavior of semiconductor devices^37–39^. The electrical transport characteristics of Schottky diodes are extensively investigated for different materials. The Schottky diode on gallium nitride (GaN) and silicon carbide (SiC) are fabricated using uniform contact of platinum (Pt) and discontinuous contact of self-assembled Au nanocrystals respectively, and demonstrated the effect of interface on SBH^40^. The nanoscale inhomogeneity of exfoliated molybdenum disulfide MoS_2_ based Schottky diode provided the information on the local resistivity of MoS_2_^41^.

In this paper, unlike conventional photodetectors, we demonstrate the effect of light irradiation on the tunneling current of gold (Au) nanotip and n-Si interface by using C-AFM. Additionally, we compare the electrical characteristics from C-AFM with the data obtained from the standard probe-station. By adapting the nanometer scale sharp AFM tip and systematically approaching the tip to the surface of n-Si we observe the prominent differences of tunneling current magnitude, in the reverse biased state of nano-Schottky diode, under the light and dark conditions. This research will pave the way for a new direction in the field of ultra-sensitive photodetectors focusing on photoresponse induced tunneling current in the nano scale Schottky junctions.

## Results and Discussions

**Figure 1:**
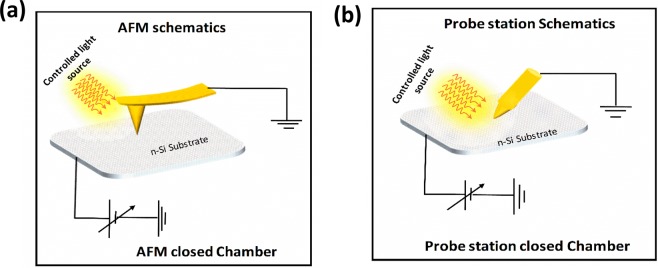
The Schematic illustration of the electrical measurement carried out by (**a**) conductive atomic force microscope and (**b**) conventional probe station.

For the revelation of electrical properties of nano-scale MS Schottky photodetector the electrical characteristics are measured by using C-AFM with different gold (Au) coated AFM tips and conventional semiconductor analyzer, KEYSIGHT B1505A. During all electrical measurements the n-Si substrate is biased, whereas the nanoscale and micron scale tips of C-AFM and probe station are kept grounded respectively, as schematically shown in Fig. 1(a,b). In order to insure the MS interface is clean and passivated the n-Si substrate is cleaned using diluted hydrofluoric acid (HF) in class 100 clean room, and then the electrical characteristics were investigated immediately, without time delay.

**Figure 2:**
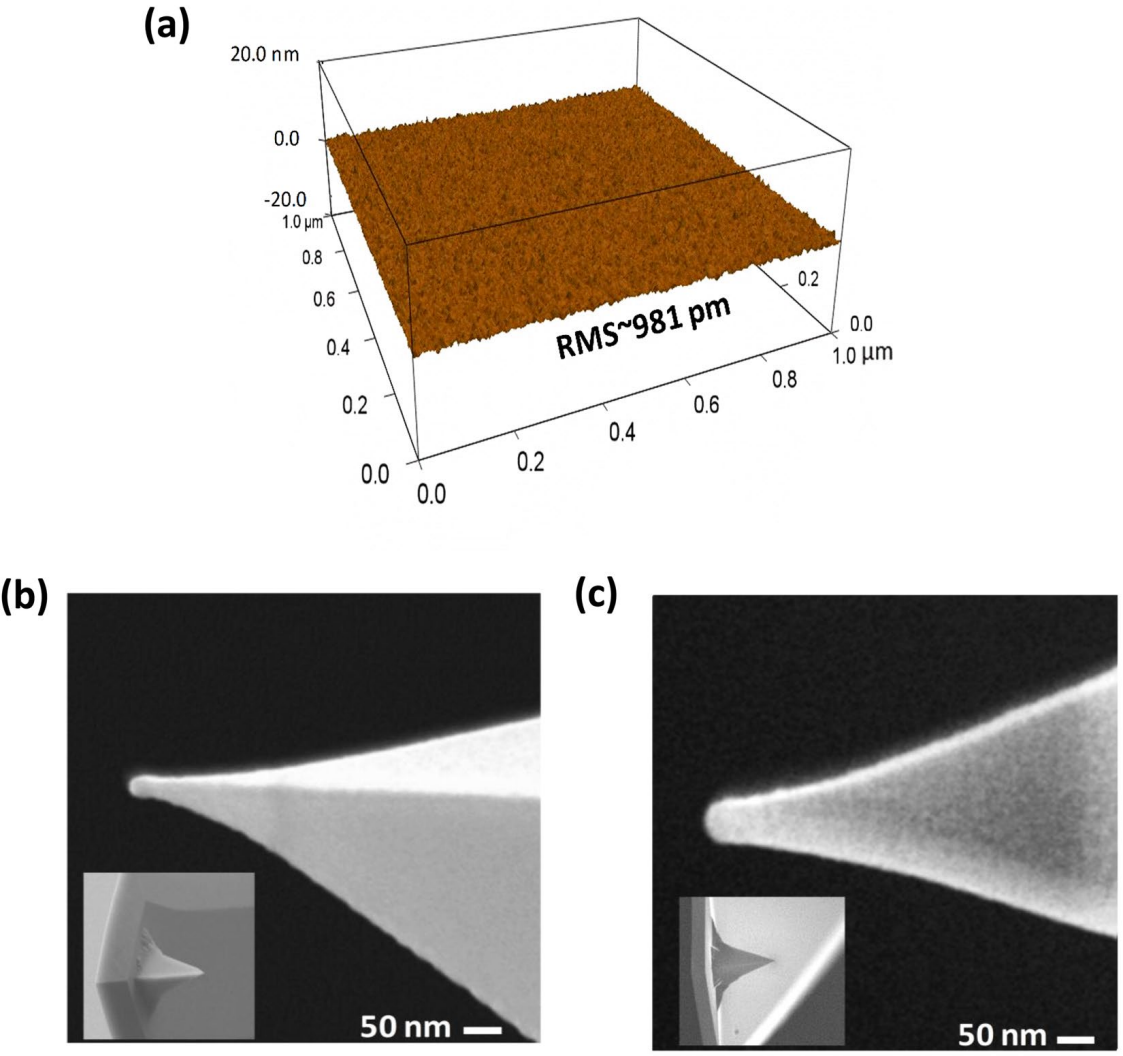
The physical characterization showing (**a**) the AFM topography of a cleaned n-Si substrate, (**b**) an SEM image of 15 nm radius tip, and (**c**) an SEM image of 30 nm tip radius. The inset in (**b**,**c**) show the SEM image of both tips in 100 µm × 100 µm frame.

The quality of the surface of n-Si substrate and the radii of the tips are explored by C-AFM topography in tapping mode and scanning electron microscope (SEM) respectively, as shown in Fig. 2. Figure 2(a) shows the highly cleaned surface of an n-Si substrate with the root-mean square (RMS) roughness of around 981 pm. This very low RMS roughness of the surface ensures the high-quality tip-semiconductor interface during the landing of the tip at the surface in the soft approach technique. Figure 2(b,c) shows the SEM micrographs of two Au coated tips with tip apex radii of 15 nm and 30 nm respectively. These different AFM tips are employed to extract the size dependent electrical characteristics of nano-scale Schottky diode and their photodetection capabilities.

**Figure 3:**
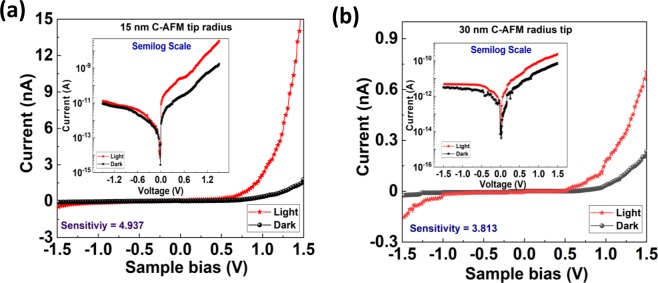
The typical electrical characteristics of the tip/n-Si Schottky diode by using (**a**) C-AFM with tip radius of 15 nm and (**b**) C-AFM with tip radius of 30 nm.

To assess the electrical characteristics of M-S Schottky diodes in the dark and light condition, we perform the (I-V) measurements in an AFM system, which is placed in a closed chamber as shown in Fig. 1(a). The sweep voltages (−1.5 V~0 ~ +1.5 V) are always applied on the substrate while the AFM tip is kept grounded. Figure 3(a,b) shows the electrical characteristics of nano Schottky diodes using Au coated tips with apex radii of 15 and 30 nm respectively. The inset of Fig. 3 shows the semilog scale plot of the electrical characteristics for better visualization of the nano-Schottky diode behavior and the photo effect.

**Figure 4:**
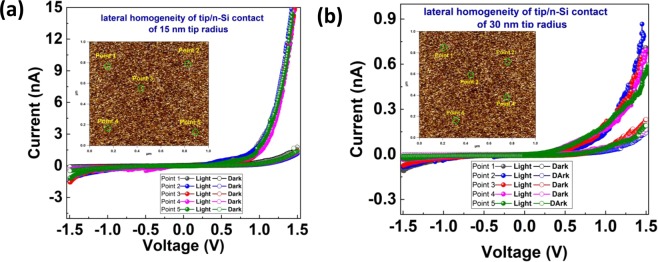
The lateral homogeneity of tip/n-Si contact and reproducibility of photo-detection phenomenon using (**a**) 15 nm tip radius and (**b**) 30 nm tip radius.

To insure the lateral homogeneity of tip/n-Si contact and reproducibility of the photo detection effect, we carried out the electrical characteristics at five different points on the surface of the substrate for both tips, in dark and light conditions. Figure 4 shows the electrical characteristics carried out at randomly chosen points. The results of Fig. 4(a,b) depict that the photo detection phenomenon is reproducible and irrespective of the location of tip/n-Si contact.

**Figure 5:**
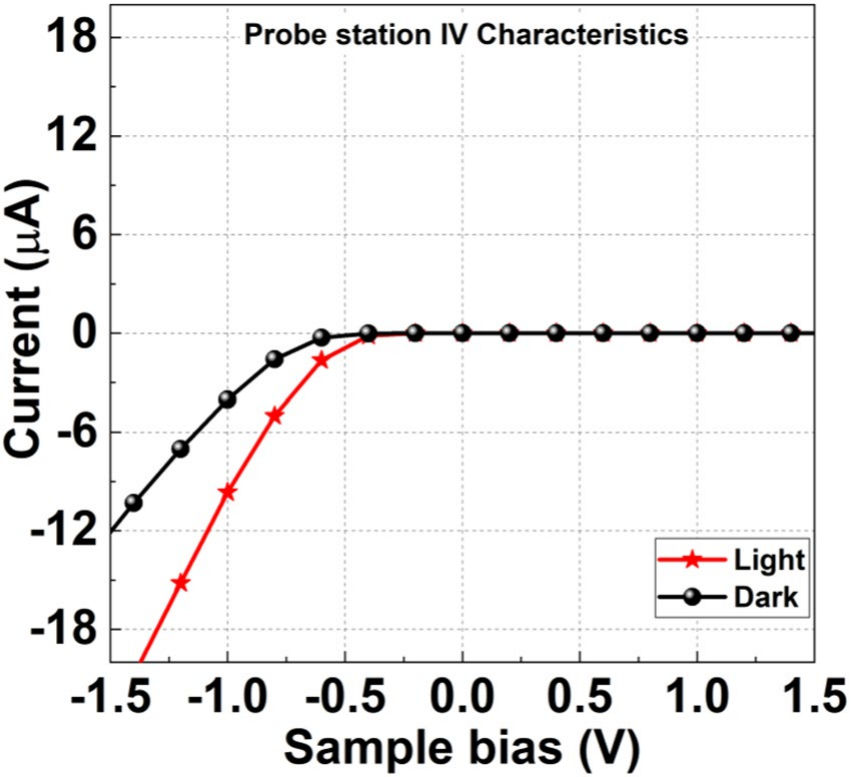
The typical electrical characteristics of the tip/n-Si Schottky diode by using conventional probe station with tip size of 20 um.

Figure 3(a,b) shows a totally opposite trend in (I-V) characteristics to conventional Schottky diodes, as we will see next in Fig. 5. When the substrate is analyzed by means of C-AFM, for the positive (reverse bias) sweep the magnitude of the tunneling current is higher than that of the negative (forward bias) sweep voltage. For comparison between the electrical characteristics of Schottky diodes and the photodetection effect at nano and micro scales, the same (I-V) measurements are conducted in a conventional Schottky diodes using normal probe station with a tip apex of ~20 µm, as shown in Fig. 5, using with the same bias conditions of C-AFM measurements. We can readily see there is a prominent difference in the electrical characteristics with light and dark conditions in the forward bias state. The magnitude of higher current for the light irradiation on the device is due to the excitation of electrons from the valence band to the conduction band of bulk n-Si.

The unconventional behavior of nano-Schottky (I-V) characteristics can be understood in the light of the fundamental electromagnetic theory, as the strength of electric field depends directly on the curvature of the conductor i.e. for the smaller curvature (smaller tip radius) the electric field generated has higher intensity and vice versa^42,43^. By considering this effect, the totally reverse trend of the I-V characteristics of the device for the conventional probe station vs. C-AFM can be well explained. When the positive voltage is applied on the substrate with respect to the AFM tip, a stronger electric field is generated at the tip-substrate interface. Due to this high intensity of electric field at the interface, the energy band bending occurs more prominently, which results in the thinning of the tunneling barrier. Thus, the smaller energy band width facilitates higher tunneling probability for electrons from the tip into n-Si substrate, leading to higher magnitude of the reverse tunneling current, for the positive sweep voltage at the substrate. This basic nano-Schottky model is detailed elsewhere^28,34^. For further understanding of the electric field enhancement at the nano-tip/Semiconductor interface, models for two probes with 15 nm and 5 µm radii are constructed using physics-based TCAD simulation to map the interface electric field.

The developed physical models in TCAD simulation uses Fermi statistics at *T* = 300 K, Shockley-Read-Hall (SRH) and Auger recombination models. Field enhancement reduces SRH recombination lifetimes in regions of strong electric fields^44^, which couldn’t be ignored at the case of the AFM tip. Therefore, the Schenk trap-assisted tunneling model^45^ was used to account for the field enhancement. Fowler–Nordheim model is included as it is assumed to occur for nonlocal tunneling at Au/Si Interfaces.

The Philips unified mobility model^46^ is used as it’s a well-calibrated model that accounts for both impurity and carrier–carrier scattering. In addition to describing the temperature dependence of the mobility, the model takes into account electron-hole scattering, screening of ionized impurities by charge carriers, and clustering of impurities. Mobility degradation due to coulomb scattering especially ionized impurities near the interface and the scattering with the surface roughness were also accounted for. Finally, the Lucent Model^47^ is used to describe mobility degradation for high electric field saturation with a charge driving force set by the gradient of a quasi-Fermi potential. Mathiessen’s rule is used to combine the doping-dependent mobility with all previously mentioned contributions.

The doping of the substrate was identical to the one used in our experimental method. The Schottky contact is defined at Au/Si material interface with barrier lowering accounted for, while the back contact defined as ohmic.

**Figure 6:**
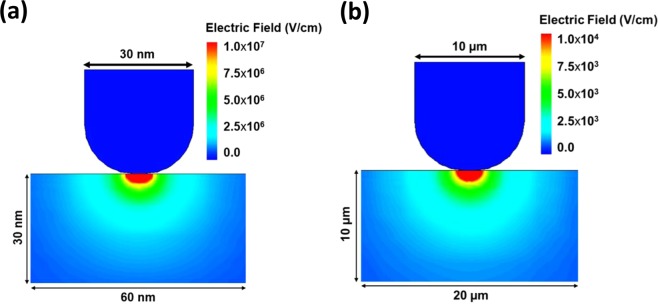
The finite element simulation of the enhanced electric distribution at (**a**) the nano probe and (**b**) the micro probe at the Si interface, at a reversed bias of +1 V and ground probe.

Both models assumed +1 V bias on the substrate and ground on the probe. In both models the electric field distribution is calculated and displayed as shown in Fig. 6(a,b). The electric field color bar scale shows the maximum electric field at the MS interface. We notice that for nano MS interface the maximum electric field is in the order of 10^7^ V/cm, which is around three orders of magnitude higher than that of the micro MS interface (in the order of 10^4^ V/cm). This extremely high electric field at the nano MS interface is responsible for the enhanced tunneling current at reverse bias for nano-probe MS measurements. We also notice that the electric field reduces at a higher rate inside the bulk than that of the micro probe case.

**Figure 7:**
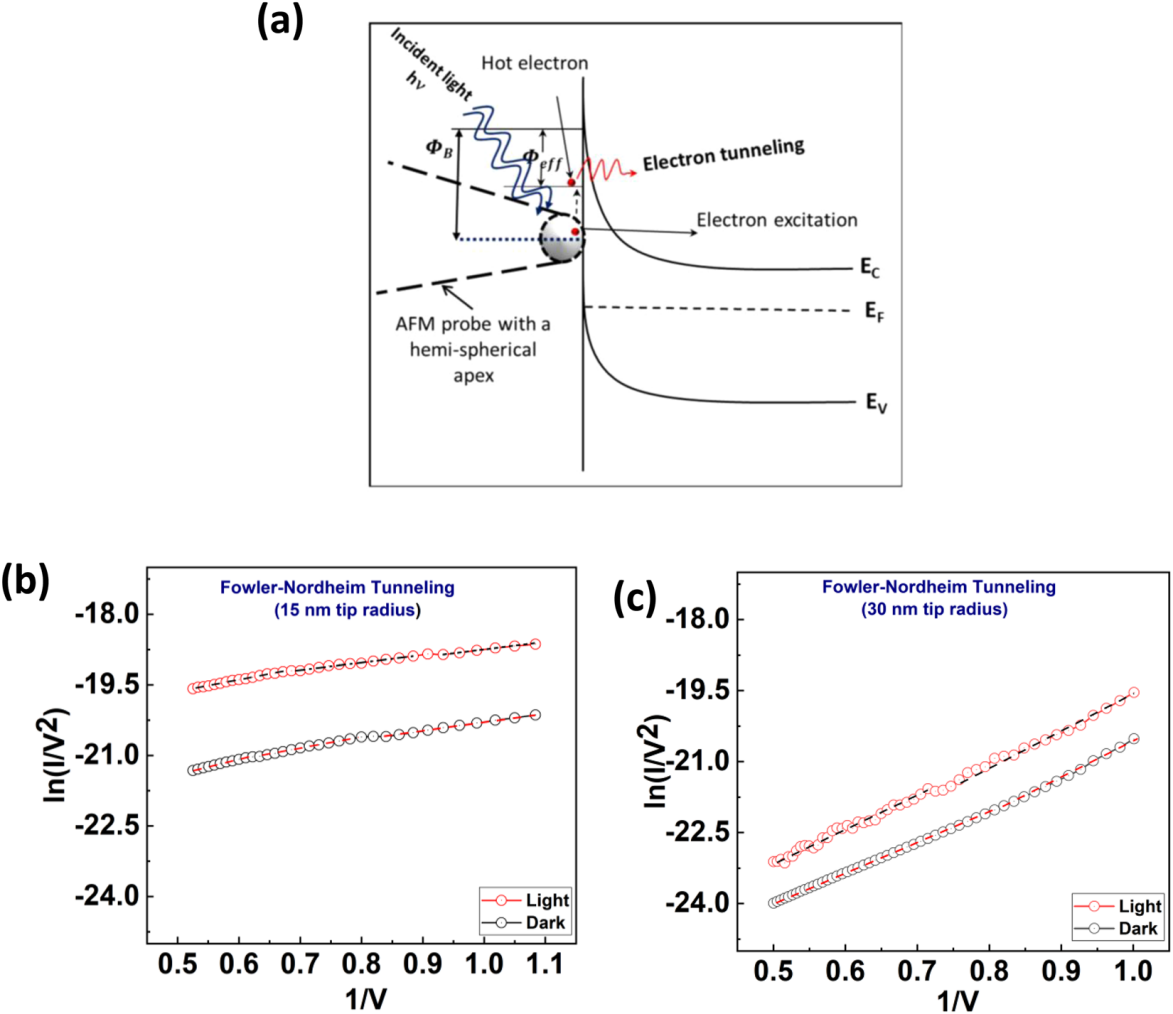
The I-V data plots according to Fowler-Nordheim theory of (**a**) A Schematic model of the energy band diagram and a photo-effect on nano-Schottky interface at a reverse bias condition. (**b**) 15 nm tip radius, (**c**) 30 nm tip radius.

To investigate the photodetection of the device, the electrical characteristics are measured in the light and dark conditions as shown in the Fig. 3. Figure 3(a,b) exhibits the higher magnitude of reverse current for the light-ON condition. In fact, the light irradiation at the interface excites the free electrons at energy level higher than the Fermi energy level at the tip apex, as shown in Fig. 7(a). Due the externally applied field at the apex these excited electrons encounter an even narrower tunneling barrier at the interface, and thus higher tunneling probability through to the conduction band in the semiconductor side. Additionally, the nanoscale metallic tip of C-AFM can be considered as a plasmonic nano-structure^48–50^, where the excited electrons due to light absorption are referred to as hot electrons. When a hot electron^51,52^ of sufficient momentum is generated by the irradiation of white light at the Schottky barrier, the high electric field stress allows the hot electron to traverse the reduced barrier leading to higher intensity photocurrent at the reverse bias.

Figure 7 quantitatively supports the tunneling phenomenon and its mechanism. As the electrical characteristics of Figs 2 and 3 revealed that the device in nanoscale regime exhibits the quantum mechanical behavior. Therefore, to quantitatively visualize the hot electron tunneling the relation between ln(I/V^2^) and 1/V are plotted for both devices dark and light conditions as shown in Fig. 7(b,c). The linear graph of ln(I/V^2^) versus 1/V indicates that the tunneling of electron in the positive sweep bias fits the field emission equation^53^. The modified nano-Schottky model that accounts for the electric photoeffect is depicted in Fig. 7(a). In conclusion, the hot electrons generated in the nanostructure i.e. nano sized tips by the incident of white light at the nano-Schottky diode interface, contribute to the photocurrent and this effect can be readily measured when the device is part of an electrical circuit, turning it to an efficient photodetector. There are several parameters that characterize the performance of the photodetectors, one of them is called sensitivity and is defined as (I_light_ − I_dark_)/I_dark_^21^_._ Interestingly, the sensitivity at the reading voltage of +1 V is calculated to be 3.1 and 4.9 for the 30 nm and 15 nm tips respectively. The higher value of the sensitivity for the smaller tip radius indicates the higher performance of detection in the smaller device size.

**Figure 8:**
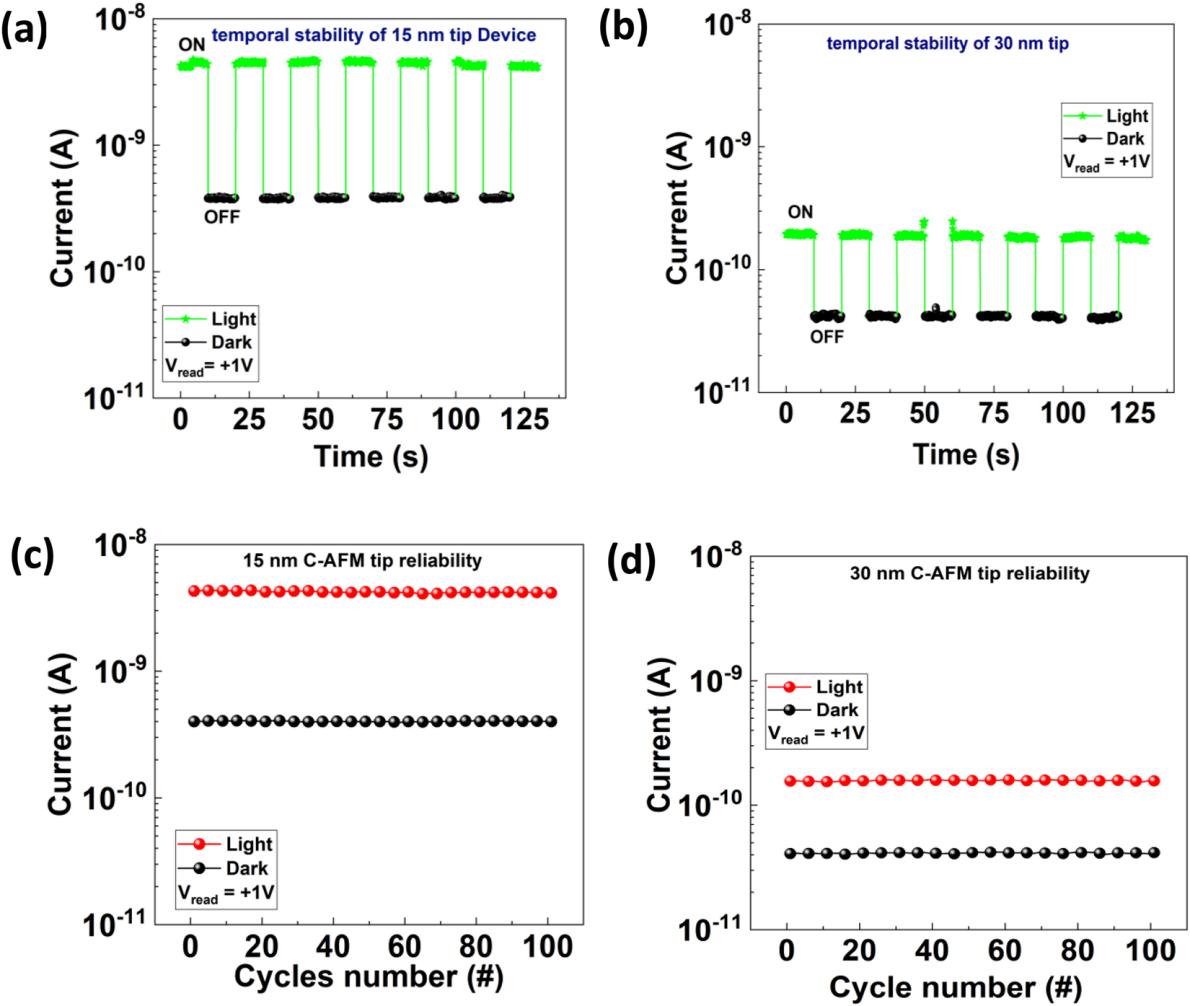
The stability and repeatable of the photo effect showing (**a**) temporal stability of 15 nm tip (**b**) temporal stability of 30 nm tip (**c**) the repeatability of 15 nm tip and (**d**) the repeatability of 30 nm tip radius.

For the industrial and practical applications of the device, the characteristics exhibited by the photo devices must be reproducible and repeatable. To assess the repeatability and reproducibility of such devices the electrical characteristics are investigated by applying a constant reading voltage of +1 V with and without the light source, as shown in Fig. 8. Figure 8(a,b) shows the temporal repeatability of the photodetection process in the devices with 15 nm and 30 nm tips respectively, in which the current is extracted in the light and dark in the intervals of 10 seconds at the reading voltage of +1 V. In this case, a constant voltage of +1 V is applied on the substrate and electric current is measured during the consecutive light pulses of 10 s width. Moreover, Fig. 8(c,d) elaborates the repeatability of the (I-V) sweeps. To confirm the repeatability of the electrical characteristics, we measure the (I-V) curves for several voltage sweeps, with and without light, and then the current values are extracted at the reading voltage of +1 V for that particular cycle. These measurements were repeated up to a 100 consecutive voltage sweeps as shown in Fig. 8 for both tips (15 and 30 nm radii). From Fig. 8 we can conclude that the process of tunneling and the photo effect is repeatable and reproducible for the nano Schottky diode with different C-AFM tips radii at sub-50 nm range. This work will attract attention for further research on nano-Schottky based photo-devices using well defined nanotips^54^.

## Conclusion

In summary we have demonstrated the photo detection effect of nanoscale Schottky diode based photodetector by using different nanotip radii of C-AFM. To insure the excellent metal/n-Si interface, the native oxide from the surface of n-Si substrate is etched out by wet etching method using diluted HF solution. The RMS roughness of 981 pm of the n-Si surface indicates a clean interface between the tip and n-Si interface. From the electrical characteristics it is observed that the tunneling current as well as the sensitivity of photodetection is higher for tips of smaller radii. The higher tunneling current for the tip of a smaller radius is attributed to the higher field intensity generated by the smaller tip radius, which in turn reduces the barrier width of tip/n-Si interface. This is tunneling current augmented by the photo effect due to generation of plasmon-induced hot-electron at the nanoparticle (i.e. C-AFM tip)/n-Si interface. Finally, the reproducibility and temporal stability of such photodetection devices have also been demonstrated.

## Experimental Approach and simulation

The n-type silicon (n-Si) wafer of resistivity 5 Ω.cm is chosen initially to study the nano-Schottky photo detection effect, using conductive atomic force microscope (C-AFM) tip on n-Si surface. The native silicon oxide from the top surface of n-Si is removed by soaking the substrate in hydrofluoric acid (HF) solution for 3 minutes. After removing the substrate from HF solution, it is rinsed in running de-ionized (DI) water. The clean surface from native silicon oxide is evident from the hydrophobic nature of the n-Si during the rinsing process. This clean surface was confirmed with the AFM topographic images. For the electrical measurements and topography of the n-Si substrate gold (Au) coated AFM tips of 146 kHz resonance frequency and force constant of 1.2–29 N/m are chosen.

For the Asylum Research MFP-3D and ORCA C-AFM systems probe holder (908.036) is capable of measuring currents from ~1 pA to 20 nA, with sensitivity of 2 nA/V and a pre-amplifier output gain resistance R of 500 M Ω. The topographic image of n-Si substrate is carried out in the AC mode imaging of AFM, with the scan length of 1 µm × 1 µm and scan frequency of 0.5 Hz. The bias was applied on the back of the n-Si substrate. To make an ohmic contact, the scratched back of the n-Si substrate is attached to a stainless steel (alloy 430) using silver paint.

The JEOL Scanning Electron Microscope (SEM) is used in order to obtained the SEM micrographs of the gold (Au) coated AFM tips. For the conventional electrical characteristics, SEYSIGHT B1505A, semiconductor analyzer is used. During the all electrical measurements the tips were grounded and a bias was applied on the n-Si substrate. Furthermore, a physics-based TCAD Sentaurus device simulation has been used to map the electric field enhancement under a nano and micro tip. Two models were constructed using TCAD simulations. The first MS model considers a nano probe of a 15 nm radius, whereas the second model considers a micro probe of a 5 µm radius. For both models an n-type silicon (Si) substrate with a resistivity of 5 Ω.cm is considered. In the simulations, a ground contact is selected on the metal probe and the substrate is biased at +1 V.
