# Author Correction: Photodetection Characteristics of Gold Coated AFM Tips and n-Silicon Substrate nano-Schottky Interfaces

**DOI:** 10.1038/s41598-020-75488-6

**Published:** 2020-10-23

**Authors:** Yawar Abbas, Ayman Rezk, Fatmah Alkindi, Irfan Saadat, Ammar Nayfeh, Moh’d Rezeq

**Affiliations:** 1 Department of Physics, Khalifa University, Abu Dhabi, 127788 UAE; 2 Department of Electrical Engineering and Computer Science, Khalifa University, POB 127788, Abu Dhabi, UAE

Correction to: *Scientific Reports* 10.1038/s41598-019-49908-1, published online 19 September 2019

Fatmah Alkindi was omitted from the author list in the original version of this Article. This has been corrected in the PDF and HTML versions of the Article.

The Author Contributions section now reads:

"M. Rezeq conceived the idea and supervised the experimental work and paper writing. Y. Abbas and A. Rezk performed the sample preparation, electrical and physical characterization along with the manuscript drafting. F. Alkindi performed data analysis and characterization. A. Nayfeh and I. Saadat contributed to data analysis and manuscript editing. All authors reviewed the manuscript."

